# Predicting the direct and indirect impacts of climate change on malaria in coastal Kenya

**DOI:** 10.1371/journal.pone.0211258

**Published:** 2019-02-06

**Authors:** Phong V. V. Le, Praveen Kumar, Marilyn O. Ruiz, Charles Mbogo, Ephantus J. Muturi

**Affiliations:** 1 Department of Civil and Environmental Engineering, University of Illinois, Urbana, IL 61801, United States of America; 2 Faculty of Hydrology Meteorology and Oceanography, Vietnam National University, Hanoi, Vietnam; 3 Department of Atmospheric Sciences, University of Illinois, Urbana, IL 61801, United States of America; 4 Department of Pathobiology, University of Illinois, Urbana, IL 61802, United States of America; 5 Malaria Public Health Research, Kenya Medical Research Institute - Wellcome Trust Research Programme, Nairobi, Kenya; 6 Crop Bioprotection Research Unit, Agricultural Research Service, U.S. Department of Agriculture, Peoria, IL 61604, United States of America; Helmholtz Centre for Ocean Research Keil, GERMANY

## Abstract

**Background:**

The transmission of malaria is highly variable and depends on a range of climatic and anthropogenic factors. This study investigates the combined, i.e. direct and indirect, impacts of climate change on the dynamics of malaria through modifications in: (i) the sporogonic cycle of *Plasmodium* induced by air temperature increase, and (ii) the life cycle of *Anopheles* vector triggered by changes in natural breeding habitat arising from the altered moisture dynamics resulting from acclimation responses of vegetation under climate change. The study is performed for a rural region in Kilifi county, Kenya.

**Methods and findings:**

We use a stochastic lattice-based malaria (SLIM) model to make predictions of changes in *Anopheles* vector abundance, the life cycle of *Plasmodium* parasites, and thus malaria transmission under projected climate change in the study region. SLIM incorporates a nonlinear temperature-dependence of malaria parasite development to estimate the extrinsic incubation period of *Plasmodium*. It is also linked with a spatially distributed eco-hydrologic modeling framework to capture the impacts of climate change on soil moisture dynamics, which served as a key determinant for the formation and persistence of mosquito larval habitats on the land surface. Malaria incidence data collected from 2008 to 2013 is used for SLIM model validation. Projections of climate change and human population for the region are used to run the models for prediction scenarios.

Under elevated atmospheric CO_2_ concentration ([CO_2_]) only, modeled results reveal wetter soil moisture in the root zone due to the suppression of transpiration from vegetation acclimation, which increases the abundance of *Anopheles* vectors and the risk of malaria. When air temperature increases are also considered along with elevated [CO_2_], the life cycle of *Anopheles* vector and the extrinsic incubation period of *Plasmodium* parasites are shortened nonlinearly. However, the reduction of soil moisture resulting from higher evapotranspiration due to air temperature increase also reduces the larval habitats of the vector. Our findings show the complicated role of vegetation acclimation under elevated [CO_2_] on malaria dynamics and indicate an indirect but ignored impact of air temperature increase on malaria transmission through reduction in larval habitats and vector density.

**Conclusions:**

Vegetation acclimation triggered by elevated [CO_2_] under climate change increases the risk of malaria. In addition, air temperature increase under climate change has opposing effects on mosquito larval habitats and the life cycles of both *Anopheles* vectors and *Plasmodium* parasites. The indirect impacts of temperature change on soil moisture dynamics are significant and should be weighed together with the direct effects of temperature change on the life cycles of mosquitoes and parasites for future malaria prediction and control.

## Introduction

The ecology of malaria is markedly complex, involving two different replication cycles of the *Plasmodium* parasite alternating in human hosts and *Anopheles* vectors [1, 2]. For malaria intervention, the ability to access health care and efficient vector control measures is critical to block the transmission and break the life cycle of *Plasmodium*. For example, the use of insecticide treated nets (ITNs) is one of the most powerful interventions that reduced malaria-related mortality from all causes among children under fives by 20% for sustained periods in Africa [3]. Climatic factors especially air temperature and humidity play an important role in malaria transmission through influence on mosquito abundance, development, biting rate, and survival as well as parasite survival and extrinsic incubation period (EIP), the time taken by the parasites to complete their sporogonic cycles [4–6]. Therefore, climate change will likely affect the dynamics of malaria and other mosquito-borne diseases in the future [7, 8].

The impacts of global warming on malaria transmission, however, remain a subject of intense debate [9, 10]. A number of studies have shown that observations of global malaria declined over the 20*^th^* century [11, 12] and the local resurgence of this disease in East Africa highlands were mainly owed to anthropogenic factors, i.e socio-economics and disease surveillance systems, rather than climatic drivers [13–15]. Conversely, other studies have suggested a close association between changes in malaria incidence and climate variability in East Africa highlands [5, 16]. Moreover, several works have reported evidence for the increase in the altitude of malaria distributions in warmer years and predictions of widespread increases and geographical shifts in distribution of malaria in the highlands of Africa and South America under climate change [17–23]. So far, most studies analyzing malaria-climate change relations have tended to consider environmental drivers and anthropogenic factors independently [24]. In a recent study, Béguin *et at*. [25] used statistical modeling to show the opposing effects of warming climate and socio-economic development on the global distribution of malaria. While malaria risk could be limited by economic growth, changes in climate suitability in poor areas remain a big challenge for malaria control [24].

At sub-regional scales, biological and mechanistic models are often used to separate out and investigate the impacts of global warming on malaria risk. Most of these models have incorporated the relationships between temperature and malaria transmission that are relatively well understood. For instance, air temperature is used to estimate the developmental rates of *Anopheles* mosquitoes in aquatic stages [26, 27] and *Plasmodium* parasites within the mosquitoes [28, 29]. Few malaria models have attempted to incorporate the causal relationship among rainfall, humidity, soil moisture, and mosquito larval habitats [17, 30–33]. The processes by which rainfall is partitioned into infiltration and stagnant water pools suitable for *Anopheles* breeding are strongly dependent on rainfall patterns, micro-topographic features, soil characteristics and vegetation cover. Nevertheless, existing models that have been used are limited in their ability to capture the changes in ecohydrological responses induced by vegetation acclimation under elevated [CO_2_] and temperature change [34–38]. These acclimatory responses have been shown to affect soil moisture and the persistence of ponding through changes in evapotranspiration (ET) [38–40], and thus habitat structure and distribution for *Anopheles* mosquitoes.

The goal of this study is to investigate the combinatorial impacts of climate change on malaria transmission dynamics through modifications in the: (i) sporogonic cycle of *Plasmodium* induced by air temperature increase, and (ii) gonotrophic cycle of *Anopheles* vector triggered by changes in the natural breeding habitat arising from the acclimatory responses of vegetation under elevated [CO_2_]. We hypothesize that changes in ecohydrologic fluxes induced by vegetation acclimation will affect vector habitat structure and distribution, thus influencing the vector abundance and malaria dynamics. Malaria transmission will be further complicated by air temperature change that affect moisture fluxes, life cycle of *Anopheles* vectors, and sporogonic cycle of *Plasmodium* parasites. Our hypothesis is tested through the use of a lattice-based malaria model in a coastal area in Kenya, East Africa at sub-regional scales. We address the uncertainty of malaria transmission in response to climate change in this region through a meta-population and stochastic modeling approach.

## Materials and methods

### Model description

**Fig 1:**
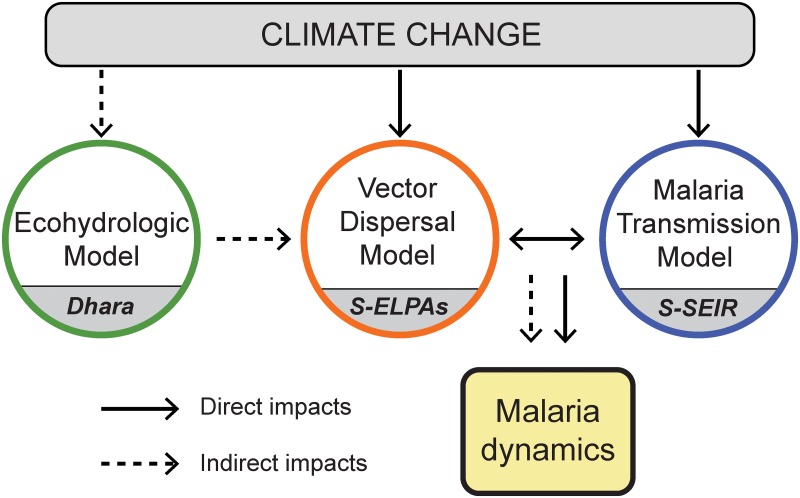
Schematic of SLIM model for predicting malaria dynamics under climate change. SLIM consists of two time-continuous space-discrete models (S-ELPAs and S-SEIR) that considered the nonlinear relationship between temperature and *Plasmodium* development inside *Anopheles* vectors for estimating the EIP. SLIM is linked with an ecohydrologic modeling framework (*Dhara*) to incorporate the acclimatory responses of vegetation on soil moisture and breeding habitat of vectors under climate change. Solid arrows represent direct impacts of temperature increase on malaria. Dash arrows represent the indirect impacts of ecohydrologic acclimation under climate change on malaria.

We use a stochastic lattice-based integrated malaria (SLIM) model [41, 42] to predict the dynamics of malaria under climate change. SLIM consists of two time-continuous space-discrete models (S-ELPAs and S-SEIR) that considered the nonlinear relationship between temperature and *Plasmodium* development inside *Anopheles* vectors for estimating the EIP [28, 29, 43]. The model incorporates explicit coupling between entomological and parasitological processes, epidemiological statuses, and hydro-climatic conditions to capture the dynamics of malaria (See Fig 1). The stochastic entomological model resolves the life cycles in aquatic and adult phases of *Anopheles* mosquitoes using the ELPAs (Egg, Larvae, Pupae, and Adults) structure [44]. The coupled epidemiology-parasitology model simulates stochastically the circulation of *Plasmodium* parasites in human hosts and adult *Anopheles* vectors using the well-known SEIR (Susceptible, Exposed, Infected, and Recovered) approach [45, 46]. These models are iteratively coupled in discrete space domains through the equality constraint of adult vector populations. SLIM also incorporates a meta-population approach to describe the spatial movements of vectors among discrete geographic domains. We refer the reader to original work [41] for detailed formulation and description of SLIM model.

Climate-driven hydrological changes under global warming are considered explicitly in SLIM for capturing mosquito’s habitat distribution. Specifically, SLIM is linked with an ecohydrologic modeling framework (*Dhara*, see ref [40]) to incorporate the acclimatory responses of vegetation on soil moisture and breeding habitat of vectors under climate change. The *Dhara* framework includes a well-tested canopy process model (MLCan, see refs [39, 47, 48]) and a benchmarked physically-based surface-subsurface flow model coupler (GCS-flow, see ref [49]) designed for capturing the coupled dynamics of moisture transport on the land surface and in the below-ground systems. It incorporates vegetation acclimation to elevated [CO_2_] and the retention of moisture flow dynamics associated with microtopographic variability. This integration provides the predictive capability to capture the impacts of environmental changes on the formation and persistence of breeding habitat.

### Study area

**Fig 2:**
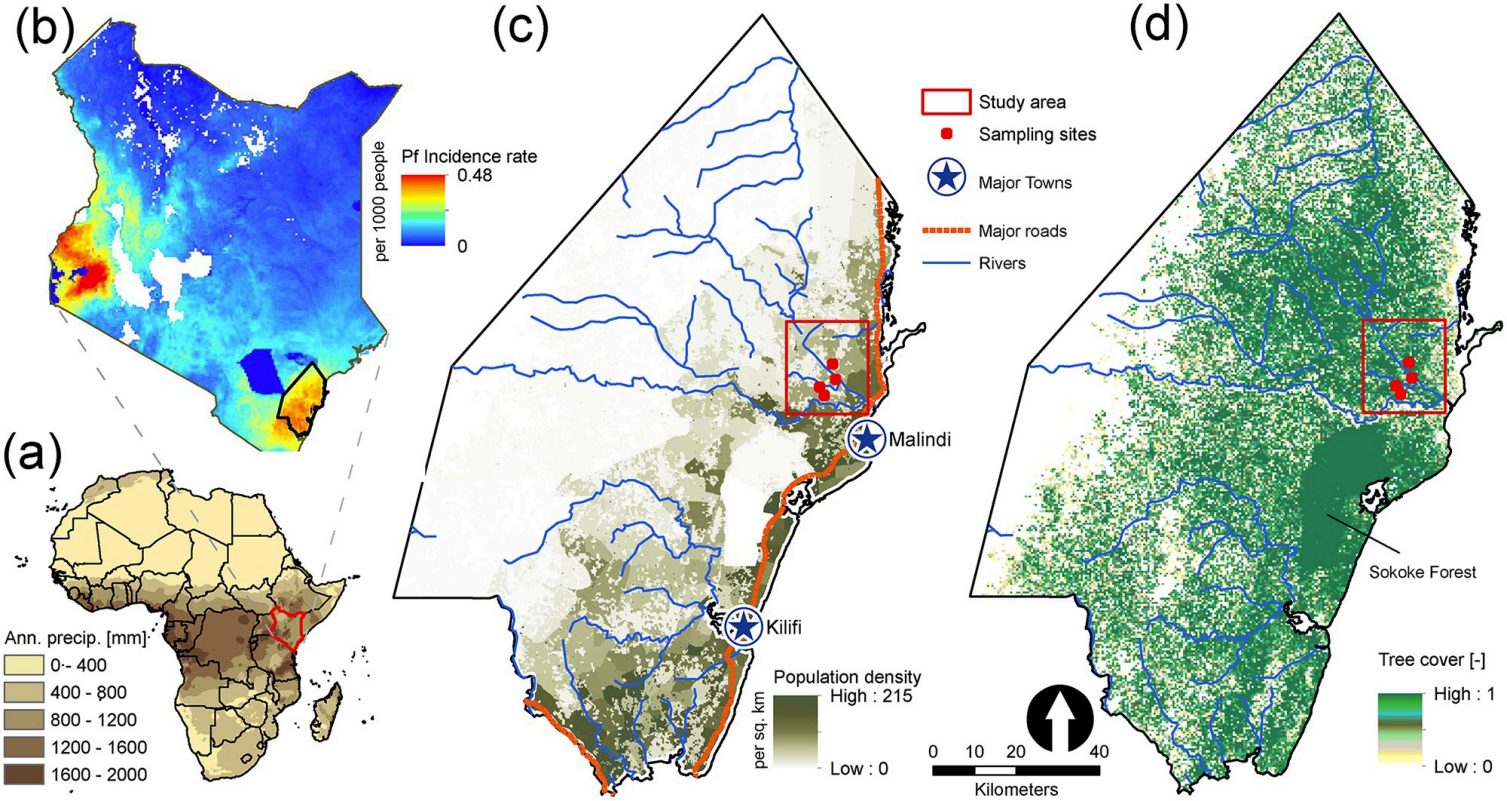
Map of study area in Kilifi county, Kenya. (a) Mean annual precipitation gradient map of Africa showing Kenya in red region. (b) Distribution of *P. falciparum* incidence rate in Kenya. Areas that have no data are shown in white. The Kilifi county (bottom right, black polygon) is one of the regions of highest malaria incidence in Kenya. Data is obtained from the Malaria Atlas Project [54]. (c) Map of population density distribution in Kilifi county, Kenya (Data is adapted from [56]). Simulations are conducted for the area of 440 km^2^ indicated by the red rectangle. (d) Map of tree cover in Kilifi, Kenya. Data is obtained from the World Resource Institute (http://www.wri.org).

The case study is conducted for a site in rural area northwest of Malindi town (3.22°S, 40.12°E), Kilifi county, Kenya (see Fig 2). The region has high levels of malnutrition and incidence rate of *P. falciparum* parasites in which the *An. gambiae* s.l. complex are the main vectors (87-95% vector population) [50]. For simplicity, we consider this complex as a single species and the only vector in the model for simulations. The average daytime temperature varies between 28°C and 34°C and mean annual precipitation is approximately 1180 mm divided into a long (April—July) and short (November—December) rainy seasons. The dominant livelihood is subsistence farming with 84.5% of the population living below the poverty line (http://www.crakenya.org/county/kilifi). Maize is the main agricultural crop for a majority of households in the region. Other crops most commonly inter-cropped with maize include millet, cassava, and beans [51, 52]. Here, we assume that maize is the dominant vegetation in each grid point in the ecohydrologic *Dhara* model. In rural areas, we also assume that natural water on the ground is the primary habitat of *Anopheles* mosquitoes. Previous studies [53] and larval collection in the region have shown that open, natural habitats were the most productive sites for *An. gambiae* larvae (see S1 Fig). The domain of simulation covers approximately 440 square kilometers (22 km north to south and 20 km east to west) with a medium to high percentage cover of vegetation. We use model parameter sets similar to those used in previous studies [27, 44, 46] for predicting malaria under climate change.

### Data

#### Malaria

Observations of malaria incidence are collected from three elementary schools (Burangi, Majahani, and Mumagani) in the study area and obtained from the Malaria Atlas Project (MAP) from 2008 to 2013 for model validation [54]. Blood samples collected from the participants are tested for malaria parasites using microscopy or rapid diagnostic tests (RDT).

#### Meteorological data

Three-hourly meteorological data from ERA-interim global reanalysis [55] by European Centre for Medium-range Weather Forecasts (ECMWF) are obtained for the study region (http://www.ecmwf.int/research/era). Data collected from 2006 to 2014 are used for baseline scenario simulations.

#### Ecophysiological data

Leaf area index (LAI) data at an 8-day interval are obtained from the Moderate Resolution Imaging Spectroradiometer (MODIS) satellite images for ecohydrologic modeling. Key model parameters in the canopy and root systems of maize crop used for the model are chosen from a prior study [47].

#### Topography and soil

**Fig 3:**
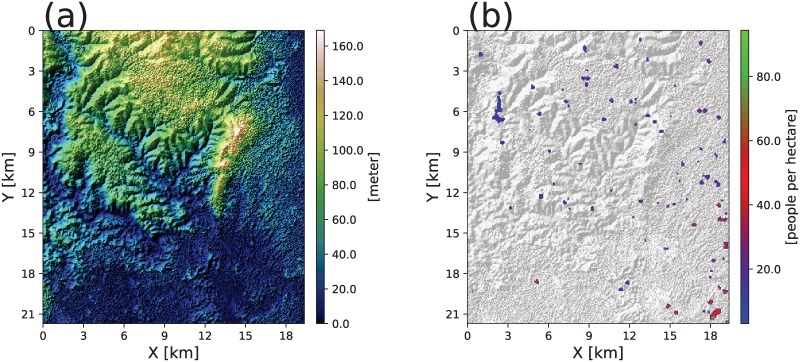
Domain of simulations in the study area. (a) Variation in topographic elevation (source: ASTER DEM). (b) Map of distribution of human population in the study area (source: [56]). The gray background represents hillshaded topography.

Topographic data at 30m × 30m resolution from the Advanced Spaceborne Thermal Emission and Reflection radiometer (ASTER) global digital elevation model are used for modeling surface ponding and runoff and belowground soil moisture dynamics. The study area elevation varies from 5 to 179 [*m*] above mean sea level (Fig 3a). Soil properties and characteristics in the area are obtained from the International Soil Reference and Information Centre (ISRIC, http://www.isric.org).

#### Census data

Human population census data at 100 [*m*] spatial resolution are obtained from the population maps for low income nations [56]. The current distribution of human population in the study domain is shown in Fig 3b. It has been shown that human mobility may contribute to the infection dynamics of malaria [57–59], especially at scales that exceed the limits of mosquito dispersal. At sub-regional scale, however, we assume that human mobility is negligible or hosts are immobile for simulations because *Anopheles* mosquitoes tend to actively seek blood meals at nighttime when hosts are sleeping in houses [27, 60]. The annual population growth rate at 4.6% is applied for future population predictions in 2050 [61].

### Mosquito habitat identification

Moisture index in a particular grid cell represents water availability that serves as breeding habitat for mosquitoes. Previous studies [62, 63] have shown that soil moisture index implicitly combines multiple weather parameters and anthropogenic features to substantially improve malaria prediction. However, a large fraction of the breeding habitat is at scales that are not detectable by currently available topographic data. As a result, there is a probability that ponding exists in small-scale topographic depressions inside a particular non-saturated cell that hydrologic modeling at 30m × 30m cannot capture. To address this scale mismatch, we incorporate the fractal structure found in topographic depressions to the estimation of moisture index. Specifically, we utilize the result that topographic depressions exist at all sizes on the landscape and follow a power-law scale [64]. We use available topographic data to find the scaling relationship of topographic depressions (see ref [41] for details) in the study domain and assumed that this relationship remains unchanged at smaller scales for estimation of the moisture index. Topographic depressions and their distribution in the study area are identified using a topographic depression identification (TDI) algorithm (see details in ref [64]). Depressions can be merged or split as water level changes, which alter the level of the depressions. Specifically, depressions of higher level *l*_*m*+1_ are created when two depressions at level *l*_*m*_ and *l*_*n*_ merge (*l*_*m*_ > *l*_*n*_).

### Climate change scenarios

We run the SLIM model for current climate conditions and contrast with simulations performed under elevated [CO_2_] and air temperature increases at different magnitudes. Firstly, reanalysis forcing data obtained from ECMWF with [CO_2_] set equal to 385 ppm is used to run the model (S0—baseline). The average ERA-Interim air temperature used in S0 is approximately 0.8 ± 0.1°C above the 1720-1800 pre-industrial level [65]. Secondly, [CO_2_] is set equal to 550 ppm projected for 2050 in the region [66] but no change in air temperature is assumed for this scenario (S1). It is unlikely that S1 will happen in the future as elevated [CO_2_] will be accompanied by air temperature increase. However, scenario S1 allows us to analyze independently the impacts of increasing only [CO_2_] on malaria dynamics. Finally, increase of air temperature at 1°C and 2°C compared to S1 is also considered along with [CO_2_] set equal to 550 ppm (scenarios S2 & S3, respectively). These scenarios highlighted the joint effects of expected air temperature change from elevated [CO_2_] on malaria transmission dynamics. Summary of climate scenarios for model simulations are shown in Table 1.

**Table 1:** Climate change scenarios for model predictions.

Scenario	[CO_2_] (ppm)	Δ*T*(°C)*
S0—baseline^†^	385	0.0
S1—projected	550	0.0
S2—projected	550	1.0
S3—projected	550	2.0

*Δ*T* represents air temperature increase compared to S0.

^†^Mean temperature is 0.8 ± 0.1°C above the 1720-1800 pre-industrial level [65].

## Results

### Power-law scaling of vector habitat

**Fig 4:**
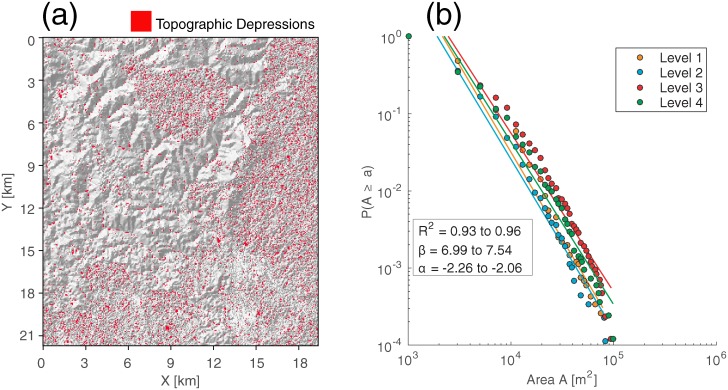
Estimation of power-law scaling of topographic depression. (a) Map of topographic depression (red polygons) identified from digital elevation model using TDI model [64]. The gray background represents hillshaded topography. (b) Scaling law relationship of topographic depressions at different ponding levels. Lines are fitted to the distributions using least square linear regression. *R*^2^, *α*, *β* represent the coefficient of determination, intercept, and slope, respectively, for each curve.

Moisture index on the land surface is expected to control vector habitat and malaria dynamics. Characterizing distribution of moisture dynamics associated with topographic depressions is thus critical to understand the life cycle of *Anopheles* vectors and the transmission of malaria under a changing environment. Fig 4 shows the distribution of topographic depressions and moisture index identified in the study area. We find that topographic depressions are ubiquitous and exist across many sizes in the area (Fig 4a, red polygons). This feature is expected to result in the heterogeneity of breeding habitat distributions and vector abundance. Fig 4b shows the exceedance probability on a log-log scale for the surface area *A* of topographic depressions found at four different levels in the study area. We fit least square linear regression lines to these distributions at each level (*R*^2^ = 0.93−0.96). The results show that the probability distribution approximates the power law fits *P*(*X* ≥ *x*) ∝ *x*^−*α*^ with slope *α* ranging from -2.26 to -2.06. Here, the slope *α* also represents the likelihood of having smaller size topographic depressions that are below the resolution observed by topographic data in the area. Water and material accumulated in these depressions provide potential habitat for mosquito’s breeding, thus affecting vector population. The mean *α* is used for moisture index estimation in the SLIM model (see details in ref [41]).

### Model validation

**Fig 5:**
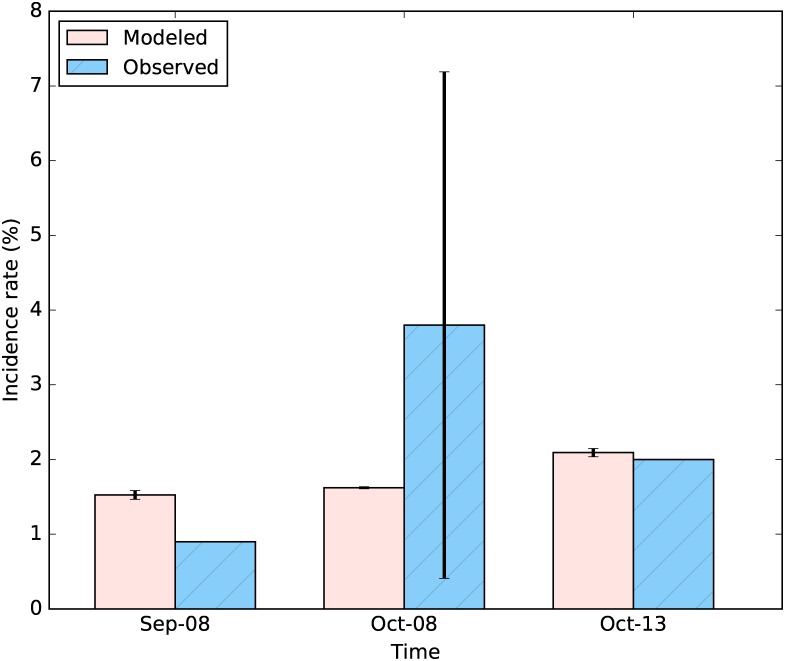
Comparison of malaria incidence rate modeled by SLIM and observed data. Vertical line represents ± standard deviation. Malaria incidence data are collected in 3 elementary schools in the area and from the Malaria Atlas Project. The high uncertainty of observed incidence in Oct 2008 comes from the variability and small sample size of the data collection.

We perform simulations for the SLIM model under the present climate condition (S0—baseline) in the study area using reanalysis data from 2006 to 2014. Comparisons of modeled and observed mean monthly malaria incidence rates (%) demonstrate the ability of the SLIM model to capture the dynamics of malaria transmission (Fig 5). We find that modeled results are in good agreement with observations measured in September 2008 and October 2013. Although a large difference is found between modeled (1.62%) and observed (3.8%) mean monthly malaria incidence rates in October 2008, modeled results remain within the range of variation of observed data.

### Ecohydrologic dynamics

**Fig 6:**
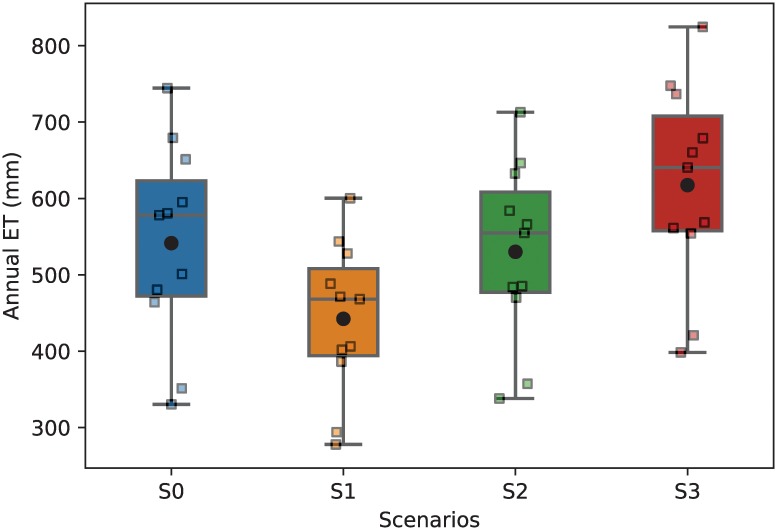
Mean annual evapotranspiration of the study region obtained from model simulations for each climate scenario. Box plots display 25^*th*^, 50^*th*^, and 75^*th*^ percentiles. Color squares represent modeled data, and black dots represent the mean value of annual ET.

We compare the mean annual ET of the study area for the [CO_2_] condition at 385 ppm (S0) with elevated [CO_2_] at 550 ppm associated with additional increase of mean annual air temperature at Δ*T*_*a*_ = 0°C (S1), Δ*T*_*a*_ = 1°C (S2), and Δ*T*_*a*_ = 2°C (S3) conditions, respectively. The boxplots showing the statistics and variability of mean annual ET for all scenarios obtained from model simulations are presented in Fig 6. The mean annual ET found in the present condition S0 is 541.1 ± 130.5 mm. We observe significant changes of annual ET, a key driver of soil moisture and ponding persistence, under all projected climate change scenarios. In S1, the decrease of annual ET (442.3 ± 100.7 mm) is attributed to the increase of vegetation water-use efficiency under the elevated [CO_2_] condition [67–69]. In S2, the mean annual ET is quite similar to that in S0. In S3, however, we find that mean annual ET is higher than in S0. These increases are directly dependent on the increases of air temperature at different magnitudes, offsetting the benefits of improving water-use efficiency under the enrichment of [CO_2_] condition. These changes in ET are expected to affect soil moisture and ponding persistence which control the habitat for *Anopheles* mosquitoes.

### Malaria transmission

**Fig 7:**
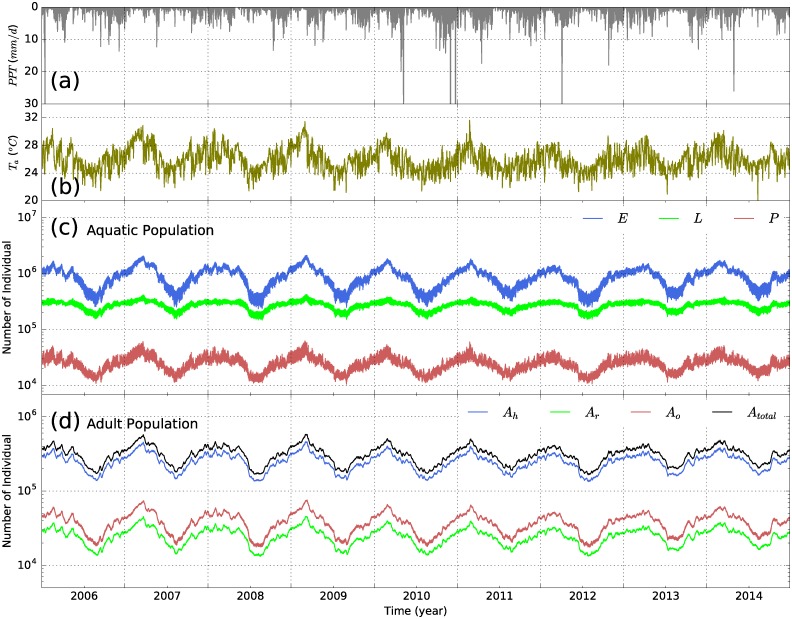
Key meteorological forcing data and variations of mosquito populations in scenario S0). (a) Daily precipitation; (b) Mean daily air temperature; (c) Population dynamics of mosquitoes in three aquatic phases (egg *E*, larval *L*, and pupal *P*) in the S-ELPAs model; and (d) Population dynamics of mosquitoes in three adult stages (host seeking *A*_*h*_, resting *A*_*r*_, and oviposition site searching *A*_*o*_) in the S-ELPAs model. *A*_*total*_ represents the sum of adult mosquitoes in all phases.

Fig 7 shows the variation of total aquatic and adult mosquito populations in the vector dispersal model (S-ELPAs) in log scales. The results show that the variation of the mosquito population in both aquatic and adult stages are highly dependent on climatic factors. The largest and smallest total mosquito population during the years are found corresponding to the highest and lowest air temperature and rainy seasons, respectively. In the aquatic stage, the sensitivity of larvae development to air temperature change is found much lower than of egg and pupae which are shown in previous studies [26]. Fig 7c implies that the mortality rate of mosquitoes subjected to environmental risks are high. The total population of *Anopheles* eggs (*E*) could be approximately 7 and 50 times higher than the larval (*L*) and pupal (*P*) populations, respectively. The population of adult *Anopheles* mosquitoes is also sensitive to climatic conditions (Fig 7d). We find that the fraction of host seeking mosquitoes (*A*_*h*_) in the adult stage is high, consisting of ∼ 70−80% of the total adult population. The sub-population of oviposition site searching mosquitoes (*A*_*o*_) or gravid females are usually 2−3 times larger than the resting mosquitoes (*A*_*r*_). The high numbers of eggs deposited by female *Anopheles* during reproduction are likely a key factor for the high density of the vector in the aquatic environment. The total number of adult mosquitoes is equal to the vector population in the Malaria Transmission Model (S-SEIR) model and play a key role in the dynamics of the disease. Simulations of malaria dynamics in present climate (S0) conditions are used to compare with results obtained from simulations under climate change scenarios discussed in the next sections.

**Fig 8:**
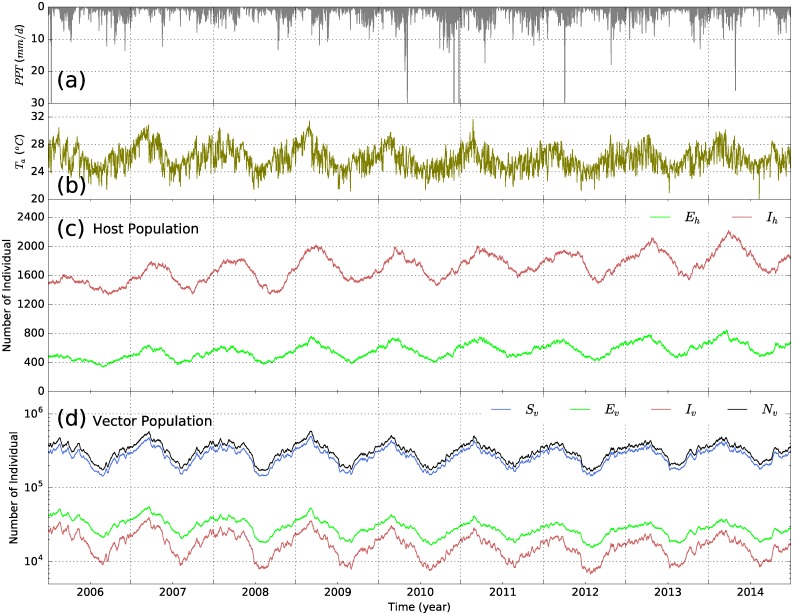
Key meteorological forcing data and the variations of malaria in scenario S0. (a) Daily precipitation and (b) Mean daily air temperature (both are the same as in Fig 6); (c) Variation of exposed (*E*_*h*_) and infectious (*I*_*h*_) host populations modeled in the S-SEIR model; and (d) Variation of vector populations (susceptible *S*_*v*_, exposed *E*_*v*_, and infectious *I*_*v*_) modeled in the S-SEIR model. *N*_*v*_ represents the total adult vectors. The S-SEIR represents different states of adult vectors shown in S-ELPAs.

The dynamics of malaria in host and mosquito populations in the study area are presented in Fig 8. The results show that, similar to the vector population, the variation of malaria incidence, including both exposed and infected cases, in the region is sensitive to climatic factors as it is directly dependent on vector density. The transmission of malaria in the study region is year-round. The largest values of exposed human cases (*E*_*h*_) are usually found after the start of the rainy season and when air temperature is high. The peaks of *E*_*h*_ are also followed after several days by the largest values of infected human cases (*I*_*h*_, see Fig 8c). During the peaks and troughs of the season, the rates of infected cases are about 2.5% and 1.0%, respectively. These results are in agreement with observations of *P. falciparum* incidence in Kilifi district shown in previous work [70].

### Impacts of climate change on malaria transmission

**Fig 9:**
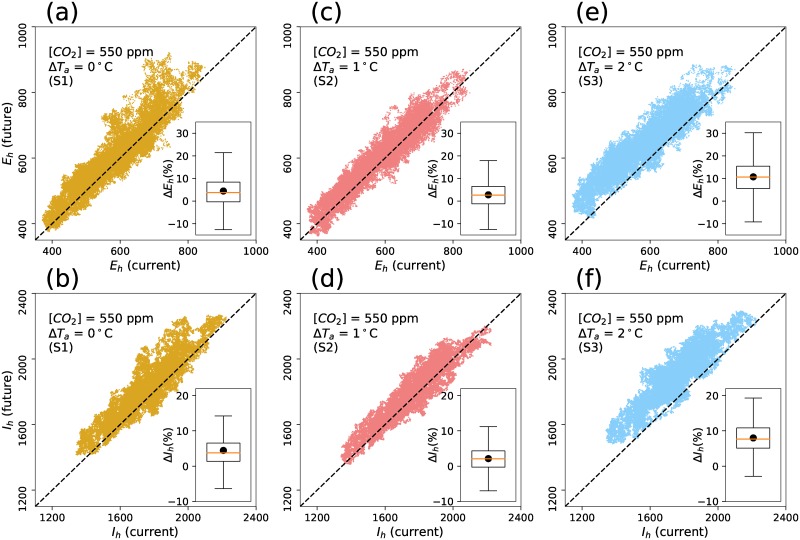
Comparison of exposed (*E*_*h*_) and infected (*I*_*h*_) malaria cases under climate change scenarios. Left column: One-to-one comparison between cases under present (S0—current) and elevated [CO_2_] (S1—future) conditions. Middle column: One-to-one comparison between cases under present (S0—current) and elevated [CO_2_] (S2—future) conditions. Right column: One-to-one comparison between cases under present (S0—current) and elevated [CO_2_] (S3—future) conditions. The inset boxplots show the difference between cases in current and future conditions. Top row shows the values of exposed cases, bottom row shows the values of infected cases. Black dots represent the mean values.

Stochastic simulations are performed to investigate the uncertainty of climate change impacts on malaria transmission. Fig 9 shows the comparison of malaria incidence (exposed and infected) between simulations under present [CO_2_] conditions (S0) and future climate change projections (S1, S2, and S3). Under the elevated [CO_2_] condition (S1), we find that the increase of soil moisture due to the changes in water-use efficiency of vegetation resulting in lowered evapotranspiration led to a higher habitat index for *Anopheles* vectors. As a result, we generally observe an increasing trend of both exposed and infected cases under S1 scenarios (Δ*E* ≈ 5%, Δ*I* ≈ 4.5%).

Under the elevated [CO_2_] condition and air temperature increase at Δ*T*_*a*_ = 1°C (S2), while increasing air temperature shortened the life cycle of both *Anopheles* and *Plasmodium*, the decrease of soil moisture and associated habitat index reduced vector abundance, thus alleviating the increase of malaria as a result of global warming. Consequently, in the S2 scenario, we generally find a small increasing trend of malaria incidence (≈ 2−3%) in comparison with present climate conditions (Δ*E* ≈ 2%, Δ*I* ≈ 3%, See Fig 9c and 9d).

In the S3 scenario, a larger increase of air temperature at 2°C leads to further reduction of soil moisture, thus causing a decrease in the habitat index for *Anopheles* mosquitoes. However, we find an increasing trend of malaria incidence under this scenario (Δ*E* ≈ 11%, Δ*I* ≈ 8%). The increase of malaria incidence found in S3 is attributed to the non-linear effects of air temperature on the life cycles of malaria vectors and parasites. In all scenarios, we find similar changing patterns in both exposed and infected cases in human population.

## Discussion

The elevated [CO_2_] condition and air temperature increase are expected to affect ecohydrological dynamics [38, 39] and nutritional quality of leaf litter that serve as food for mosquito larvae [71, 72]. Characterizing such alterations is important to understand the impacts of global warming on malaria transmission. A number of studies have shown the relationships between air temperature and precipitation changes on the dynamics of malaria [17–23]. In this work, our model incorporating the acclimatory mechanisms of vegetation under climate change is used to predict the transmission dynamics of malaria. Model results indicate the opposing effects of elevated [CO_2_] and air temperature increase on the dynamics of malaria. Given the complexity of malaria transmission under environmental disturbances, these effects would play an important role to better understand how malaria will be likely altered under climate change, thus contributing to the intervention of this disease.

Simulations obtained from projected climate scenarios show that the elevated [CO_2_] condition increases the habitat index for mosquito reproduction, which leads to higher density of vectors and an increase in malaria incidence. Unlike the elevated [CO_2_] condition, the increase of air temperature has two distinct effects on malaria dynamics. First, higher air temperature reduces soil moisture, thus decreasing the habitat index for the anopheline vector. Second, it also nonlinearly shortens the life cycles of *Anopheles* and *Plasmodium*. Under low air temperature increase, the effects of air temperature change on these life cycles are not much stronger than the impacts of soil moisture decrease on vector abundance. As a result, the trend of increasing malaria incidence is small. However, under high air temperature increase, nonlinear effects of air temperature are stronger than the impacts of soil moisture decrease on vector abundance, resulting in larger changes of malaria incidence.

The main findings from this study may shed light on better understanding the linkage between climate change and the infection dynamics of malaria. The results demonstrates that the proposed modeling approach is robust and can also be used to investigate how other changes in the natural environment affect malaria transmission. Moreover, this work can be applied to analyze the impacts of environmental changes on other mosquito-borne diseases in particular and vector-borne diseases in general.

We conclude by acknowledging several limitations of this study. First, habitat index estimated only from open topographic depressions limits the applicability of the proposed model in non-rural areas. In fact, man-made water containers and stagnant water less exposed to ET are also potential habitats for mosquitoes. However, the model can be extended using statistical techniques to analyze the uncertainty and impacts of small-size anthropogenic factors on the dynamics of malaria in urban environments. Second, the dynamics of malaria caused by multi-species vectors are not considered. Although *An. gambiae* s.l. is the main vector in the present study area, malaria transmission in many other places is influenced by population dynamics of several vector species. Finally, human hosts are assumed immobile in our model. It has been shown that human mobility may contributes to the infection dynamics of malaria [57–59], especially at scales that exceed the limits of mosquito dispersal. Further work should incorporate the dynamics of multi-species vectors and human mobility to better capture the infection dynamics of malaria.

## Supporting information

S1 Fig *Anopheles* larval samples collected at different habitat types in Kilifi district during the 2006-2012 period. A large fraction of the samples was found in open habitats (TIF).
(EPS)
